# When You Think of and Identify Yourself as a Nurse, You Will Become More Deontological and Less Utilitarian

**DOI:** 10.3390/ijerph21060712

**Published:** 2024-05-31

**Authors:** Mufan Zheng, Junhua Zhao, Xielan Zhang

**Affiliations:** Department of Psychology, Wuhan University, Wuhan 430072, China; zhangxielanpsy@whu.edu.cn

**Keywords:** moral judgments, deontology, utilitarianism, nursing role, professional identification

## Abstract

This study aims to examine how the activation of the role of nursee and professional identification as a nurse can influence moral judgments in terms of deontological and utilitarian inclinations. In Study 1, a priming technique was used to assess the impact of activating the nursing concept on moral reasoning. Participants were randomly assigned to either a nursing prime or neutral prime condition. By using a scrambled-sentence task, participants were prompted to think about nursing-related or neutral thoughts. Following the priming task, participants were asked to respond to 20 moral dilemmas. The process dissociation approach was employed to measure the degree of deontological and utilitarian tendencies in their moral reasoning. In Study 2, participants completed the nursing profession identification scale and the moral orientation scale before engaging in moral judgments similar to those in Study 1. The findings revealed that priming the concept of being a nursee resulted in an increase in deontological clinical inclinations while having no significant effect on utilitarian inclinations. Additionally, a positive correlation was observed between identification with the nursing profession and deontological clinical inclinations, whereas a negative correlation was found with utilitarian inclinations. Deliberation orientation acted as a complete mediator in the relationship between nursing professional identification and deontological tendencies and as a partial mediator for utilitarian tendencies.

## 1. Introduction

Health services are centered around the provision of care and assistance to individuals who need medical attention. Nurses are at the forefront of patient care, providing direct assistance and support to individuals in various healthcare settings [1]. Ethical judgments are of paramount importance in the nursing profession. They guide nurses in providing patient-centered care, resolving ethical dilemmas, upholding professional integrity, and building trust. By incorporating ethical considerations into their practice, nurses contribute to the overall well-being and safety of their patients while maintaining the highest standards of professionalism [2,3]. Specifically, ethical judgments can help nurses advocate for their patients’ rights and well-being. Nurses must make decisions that prioritize the best interests of their patients, ensuring they receive appropriate care, respect, and dignity [4]. Ethical judgments provide a framework for nurses to make informed decisions when faced with complex situations. They help nurses navigate moral dilemmas, such as end-of-life care, confidentiality breaches, and resource allocation, by considering the values and ethical principles involved [5]. Therefore, understanding the relationship between nursing role and ethical decision-making is pivotal.

Classical moral studies follow the rationalist approach, commonly holding the belief that moral development, moral principles, and moral reasoning styles are relatively stable features of people [6,7]. They posit that moral judgment is a conscious process, dividing moral concepts into different stages characterized by age and psychological maturity, emphasizing the role of cognition in moral judgment [8]. However, in recent decades, researchers proposed the intuitionist model. This model stressed the role of context, intuition, and emotions, and challenged the position of moral reasoning as the sole or even primary means of moral judgment [9]. The intuitionist model proposed that moral judgments are reached by various contextual, affective, and cognitive factors, rather than just the results of inner reasoning.

Some previous studies have examined the morality of nurses. These studies primarily focus on the relationship between moral judgment and the ethical behavior of nurses, measuring the maturity of nurses’ ethical development based on Kohlberg’s moral development stage model, and exploring how training and education can enhance the moral competence of nurses [1,3,10,11]. However, these studies are mostly based on the rationalist model. When considering nurses’ moral judgments, they tend to view nurses’ moral reasoning and judgments as a purely rational process, where nurses’ moral reasoning is dependent on their development of this stage of moral judgments. Few studies have investigated how the nursing profession as a contextual factor influences the moral thinking of individuals. This research aimed to examine how the concept of nursing roles and nursing professional identification influence deontological and utilitarian inclinations in ethical decisions.

### 1.1. Moral Judgment and Process Dissociation Procedure

Moral judgment is defined as evaluations (good vs. bad) of the actions or character of a person that are made concerning a set of virtues held to be obligatory by culture or subculture [9]. People often judge actions based on two principles: utilitarianism and deontology. Utilitarian judgments maximize the greatest good for the greatest number, whereas deontological judgments check whether a specific action is against existing rules, principles, and norms that must be honored [12,13].

There are two models that can be used to explain the moral judgment process: the intuitionist model and the rationalist model. Classical moral studies follow the rationalist approach (e.g., Kohlberg’s theory [6] and Piaget’s theory [14]), commonly holding the belief that moral development, moral principles, and moral reasoning styles are relatively stable features of people [7]. They posit that moral judgment is a conscious process, dividing moral concepts into different stages characterized by age and psychological maturity, emphasizing the role of cognition in moral judgment [8]. Later, intuitionist models stressed the role of context, intuition, and emotions, and challenged the position of moral reasoning as the sole or even primary means of moral judgment [9]. The intuitionist model proposed that moral judgments are reached by various contextual, affective, and cognitive factors, rather than just the results of inner reasoning.

Also, some researchers have found that the process of moral decision-making involves both rational reasoning processes and intuitive influences. Greene et al. proposed the dual-process theory of moral judgment based on this idea [15]. According to this theory, individuals’ moral cognition consists of two processing systems—intuition and cognitive reasoning—which compete in the process of decision-making and judgment [16]. The first system is automatic, intuitive, fast, and sensitive to emotions, generating an emotional response to moral dilemmas leading to support for moral rules; the second system is reflective, cognitive, slower, and more sensitive to outcomes, with judgments leaning towards utilitarianism [17]. According to Greene’s research [15,18], cognitive processing is needed to override moral inferences made by intuition to support utilitarian viewpoints. Our research is more based on the dual-process model. We believe that the context in which individuals are situated is more likely to influence their moral thinking and moral judgment.

Meanwhile, The present study followed Conway and Gawronski’s study [19] using the Process Dissociation Procedure to discuss how nursing role affects utilitarian and deontological inclinations in moral judgments. The classical approach treats utilitarian and deontological inclinations as opposite ends of a bipolar continuum. However, Conway and Gawronski proposed that utilitarian and deontological inclinations are conceptually distinct and functionally independent processes, so moral judgment can be based on both inclinations at the same time, rather than only one of them [19]. They think that classical moral dilemmas such as the footbridge dilemma cannot analyze the specific contribution of deontological inclination or utilitarian inclination in moral judgments. Therefore, they adapted the Process Dissociation (PD) procedure proposed by Jacoby [20] to study utilitarian and deontological inclinations in moral dilemma judgments. 

### 1.2. Moral Judgements of Nurses

A series of studies have focused on how nurses’ moral judgments affect their moral behavior and clinical practice, suggesting that nurses’ moral judgments play a crucial role in guiding clinical practice and ensuring patients receive quality healthcare [1,2,3,21,22]. Therefore, studying nurses’ moral judgments holds significant importance. An early study found a high correlation between moral judgments and moral behavior among practicing nurses [2]. Similarly, recent research conducted on registered nurses has also identified the same effect. The study assessed the judgments and ethical behaviors of nurses using the Hospital Ethics Committee Survey Questionnaire and found that the judgments and ethical behaviors of nurses were evaluated to be at the moderate and good levels [1].

Furthermore, researchers have emphasized the need to enhance nurses’ moral judgment abilities and moral development to better guide their professional work and provide improved healthcare services to patients [1,21]. Thus, many studies on nurses’ moral judgments have adopted Rest’s Four Component Model and Kohlberg’s Moral Development Theory to understand the moral judgments of nurses at different stages of their professional careers and to explore ways to enhance nurses’ moral judgment abilities [1,2,10,11]. 

The role of nursing education in developing ethical competence and moral judgment has been explored in various studies [1,2,10,11]. Researchers have examined the effectiveness of ethics education programs, the integration of ethics into nursing curricula, and the impact of educational interventions on nurses’ moral judgment skills. Previous studies utilized different tools to measure the impact of educational interventions on improving nurses’ moral judgment abilities, including Rest’s Defining Issues Test [2,23], the Hospital Ethics Committee Survey Questionnaire [1], the Questionnaire of Moral Judgment and Ethical Decisions (QMJED) [24], and the Moral Competence Questionnaire for Public Health Nurses [3]. These studies have found that educational interventions effectively enhance nurses’ moral judgment abilities. This effect has been observed not only among registered nurses but also among practicing nurses and nursing students. Also, these findings have been proved in different cultural contexts [1,3,10,23].

Nurses often encounter ethical dilemmas in their practice, such as end-of-life care, patient autonomy, informed consent, confidentiality, and resource allocation. Most moral judgments in dilemmas are based on two categories of moral theories: deontology and utilitarianism. However, previous research has paid limited attention to how nurses employ different moral theories to engage in moral reasoning when facing ethical dilemmas. Meanwhile, previous research on nurses’ morality has been largely based on Kohlberg’s theory of moral development stage, linking individuals’ moral development to age and experience [1,3,10,11]. However, few have considered that profession is also a context; when individuals think about and identify with the nursing profession, they are in one context, and when they are not thinking about their profession, they are in another context. These contextual differences can lead to differences in their moral reasoning. 

Therefore, the present study employs the process dissociation technique to measure nurses’ deontological and utilitarian inclinations and aims to examine how nursing profession as a context influences individuals’ moral judgments. Meanwhile, we further aim to discuss what cognitive processes contributed to this moral reasoning. We used the Moral Orientation Scale [25] to assess four moral orientations (integration, deliberation, rule, and sentiment inclinations) usually involved in moral reasoning and tested if they explain the effect of nursing-role identification on deontological and utilitarian thinking.

The dual-process model of moral judgment [26] states that there are two key psychological processes involved: affective processes and cognitive processes. A series of previous studies have demonstrated that a number of factors-associated affective processes lead to more deontological thinking and cognitive evaluation of outcomes is usually associated with utilitarian moral judgments, for example, harm and intuition [27], cognitive load [28,29], decision time [30], reasoning [31], etc. Recently researchers have also focussed on some other processes that might be involved in moral reasoning but have not been investigated by the dual-process model, such as rule orientation, deliberation orientation, integration, and sentiment orientation [25]. Previous studies found that higher rule orientation and sentiment orientation can predict higher deontological moral inclination and lower utilitarian inclination, while higher deliberation orientation is negatively associated with deontological inclination and positively associated with utilitarian inclination [15,25,26]. Integration orientation is characterized by the inclination to combine emotional reactions with cognitive reflection, which is considered a characteristic of advanced moral reasoning [32]. Individuals with high scores in integration orientation engage in a thorough consideration of pertinent arguments, exhibit emotional empathy towards all potential victims, and demonstrate flexibility in considering various response options. Usually, this produces a modest deontological pattern of responding to relative dilemma judgments [25].

We predict that nursing role will lead to a higher deontological inclination. Nursing is a profession deeply rooted in ethical principles [33]. Nurses are bound by a code of ethics, which emphasizes the importance of patient advocacy, autonomy, and respect for human dignity. These ethical guidelines encourage nurses to prioritize their duty to do what is right, regardless of the consequences. Also, nurses have a strong sense of duty and responsibility towards their patients. They are trained to prioritize patient safety, provide competent care, and act in the best interest of the patient [34]. Deontological ethics reinforces the idea that nurses have a moral obligation to fulfill their professional duties, even when faced with challenging moral dilemmas [12].

Meanwhile, nursing practice requires nurses to engage in complex decision-making that involves integrating both affective and cognitive processes [35]. Nurses must consider not only the clinical and medical aspects of a situation but also the emotional and psychosocial dimensions of patient care [36]. They often encounter morally challenging situations where they need to navigate conflicting values, emotions, and ethical considerations. The integration of affective reactions with cognitive deliberation allows nurses to thoroughly consider the potential impact of their decisions on patients, families, and other stakeholders. Previous research found that integration inclination is usually associated with higher deontological thinking [25], so we assumed that nursing role may increase deontological thinking via integration inclination.

We predict that nursing role will lead to a lower utilitarian inclination. Nurses often face ethical dilemmas in which there are no clear-cut or objectively measurable outcomes. The complexity and uncertainty of healthcare situations make it challenging to accurately predict the consequences of different courses of action [37]. In such cases, nurses may be more inclined to rely on their professional judgment, experience, and intuition rather than solely relying on utilitarian calculations [38].

Nurses frequently work in high-pressure environments with limited time and resources [39]. These constraints may impact their decision-making, leading them to prioritize immediate actions that address the patient’s immediate needs rather than considering long-term consequences. Utilitarianism often requires a more comprehensive analysis of outcomes, which may be challenging given the practical realities nurses face [12].

Nursing ethics emphasize principles such as autonomy, beneficence, and non-maleficence. These principles guide nurses in providing compassionate care and respecting patient autonomy [40]. While utilitarianism also values these principles, the emphasis on the overall outcome may conflict with the individual-focused approach that nurses are trained to uphold. 

Also, nurses may prioritize individual patient needs and immediate concerns, which may sometimes diverge from the utilitarian perspective. Nurses are trained to prioritize the well-being and individual needs of their patients [41]. This focus on patient-centered care may lead nurses to consider the immediate needs and preferences of the patient, potentially influencing their moral decision-making. In some cases, this may conflict with the utilitarian perspective, which aims to maximize overall happiness or well-being for the greatest number of people.

Therefore, we assumed that deliberation, rule, integration, and sentiment thinking orientations can explain the effect of nursing role on moral judgments. 

### 1.3. The Present Study

The present study was designed to test the effects of nursing role on moral judgments. Specifically, we manipulated the concept of nursing role (Study 1) and measured nursing-role identification (Study 2), and used the PD procedure to assess participants’ deontological and utilitarian orientations. Also, in Study 2, we assessed four moral orientations (integration, deliberation, rule, and sentiment), and tested if they mediated the effect of nursing-role identification on deontological and utilitarian thinking. We predicted that nursing-role identification would decrease the utilitarian inclination during moral reasoning and increase the tendency to adopt a deontological approach, and that deliberation, rule, integration, and sentiment orientations mediated the relationship between money and utilitarian and deontological moral thinking inclinations. 

## 2. Study 1

### 2.1. Methods

Participants and Design: We recruited 90 nurses (77 female and 13 male, aged from 18 to 50, *M* = 26.86, *SD* = 7.24) from 2 hospitals in China. Participants were randomly assigned to one of two conditions: nursing role prime or neutral prime. All subjects participated voluntarily.

Procedure and Materials: Participants firstly were asked to complete a scrambled-sentence task in which they were exposed to nursing-role-related words or neutral words. The scrambled-sentence task consists of 30 sets of five jumbled words. Participants had to create a sensible sentence using four of the five words within the time limit. In the neutral condition, the phrases primed neutral concepts (e.g., words “hometown, future, occasionally, he, recall” can be used to create a sentence “he occasionally recalls hometown”). In the nursing role prime condition, 15 of the phrases primed the concept of nursing role (e.g., words “in, observe, nurses, work, hospital” can be used to create a sentence “nurses work in hospital”), whereas the remaining 15 sets of words were neutral phrases.

After the scrambled-sentence task, participants read 20 moral dilemmas in the study of Conway and Gawronski, in which they must choose whether to perform a harmful action to achieve a particular outcome [19]. After reading each dilemma, participants were asked to indicate whether the described action would be *appropriate* or *inappropriate* according to their opinion. There were 10 basic dilemmas in total, each being presented in incongruent and congruent versions. Incongruent dilemmas depict the outcomes of harmful action as more beneficial than the harm caused by the act. Responses consistent with utilitarianism (accept harm) are incongruent with responses consistent with deontology (reject harm). In congruent dilemmas, the outcome of causing harm is diminished such that causing harm no longer maximizes outcomes, so responses consistent with utilitarianism are congruent with responses consistent with deontology. Finally, participants provided demographic data.

### 2.2. Results and Discussion

Overall, harmful action was judged acceptable in 50.1% of the incongruent dilemmas and 37% of the congruent dilemmas. The difference between the two kinds of dilemmas was statistically significant, *t*(89) = 9.81, *p* < 0.001.

PD scores of deontology (D parameter) and utilitarianism (U parameter) were calculated using the algebraic formulas of Conway and Gawronski’s study [10]. We used ANOVA analysis to test the effect of nursing role on D and U parameters, and participants in the nursing role group (*M* = 0.61, *SD* = 0.18) showed higher deontological inclinations compared to those in the control group (*M* = 0.53, *SD* = 0.15), *F*(1, 88) = 5.33, *p* = 0.02, *η*^2^*_p_* = 0.06, but there was no significant difference between the nursing role group (*M* = 0.12, *SD* = 0.13) and control group (*M* = 0.15, *SD* = 0.13) in utilitarian inclinations, *F*(1, 88) = 1.31, *p* = 0.26, *η*^2^*_p_* = 0.02.

Priming the role of nurse affected deontological inclinations but did not influence utilitarian inclinations. Participants primed with nursing role showed higher deontological inclinations than those primed with a neutral concept. This is different from our assumption. This may be due to the scrambled-sentence task not being able to truly evoke the nurses’ associations with real work contexts. Therefore, in Experiment 2, we directly measured the identification with nursing role, aiming to explore the long-term influencing traits associated with professional role on moral judgments.

## 3. Study 2

Study 2 investigated the relationship between nursing-role identification and moral judgments to further test whether long-term traits associated with nurses’ professional role have a similar effect on moral thinking as priming the role of nurse. We hypothesized that individuals with higher levels of nursing-role identification are more likely to rely on deontological thinking and less likely to rely on utilitarian thinking in moral judgments. Meanwhile, to unveil the mechanism underlying the effect of nursing role on moral thinking, we also used the moral orientation scale [25] to assess participants’ integration, deliberation, rule, and sentiment orientations and tested if these orientations mediate the effect of nurse-role identification on deontological and utilitarian approach. 

### 3.1. Methods

Participants: We recruited 496 subjects (193 male and 303 female, aged from 18 to 59, *M* = 29.58, *SD* = 8.10) from an online platform (www.wenjuan.com, accessed on 5 July 2021) in China. All subjects participated voluntarily, and none of them participated in Study 1. 

Procedure and Materials: In this study, nursing-role identification scale (α = 0.736) is from the organizational identification scale originally developed by Mael and Ashforth [42] with the word ‘firm’ substituted by ‘profession’ in the scale items. We used the five-item edition modified by Lui, Ngo, and Tsang [43]. The 5-item scale is rated on a 7-point scale ranging from 1 (strongly disagree) to 7 (strongly agree). 

Next, participants were instructed to complete the Moral Orientation Scale (MOS), consisting of 28 items, and each of the 7 items within each subscale [25]. Four subscales measure integration, deliberation, rule, and sentiment. These four orientations are all important thinking processes in moral reasoning. Participants indicated their level of agreement on a 7-point Likert scale, ranging from strongly disagree (1) to strongly agree (7). The order of the items was randomized. The internal consistencies of the subscales were found to be acceptable to good, with Cronbach’s alpha coefficients ranging from 0.73 to 0.89. 

After answering questions on nursing-role identification scale, participants read moral dilemmas and made moral judgments as in Study 1. Finally, participants provided demographic data.

### 3.2. Results and Discussion

Overall, harmful action was judged acceptable in 52.8% (*SD* = 0.19) of the incongruent dilemmas and 37.9% (*SD* = 0.18) of the congruent dilemmas. The difference between the two kinds of dilemmas was statistically significant, *t*(495) = 18.00, *p* < 0.001.

PD scores of deontology (D parameter) and utilitarianism (U parameter) were calculated using the algebraic formulas of Conway and Gawronski [19]. We used regression analysis to examine the relationship between nursing-role identification and deontological inclination and utilitarian inclination, and found that nursing-role identification was positively related to individuals’ deontological inclinations, *β* = 0.20, *t*(495) = 4.44, *p* < 0.001, 95%CI [0.02, 0.04], and was negatively associated with individuals’ utilitarian inclinations, *β* = −0.10, *t*(495) = −2.21, *p* = 0.027, 95%CI [−0.03, −0.002]. 

**Figure 1:**
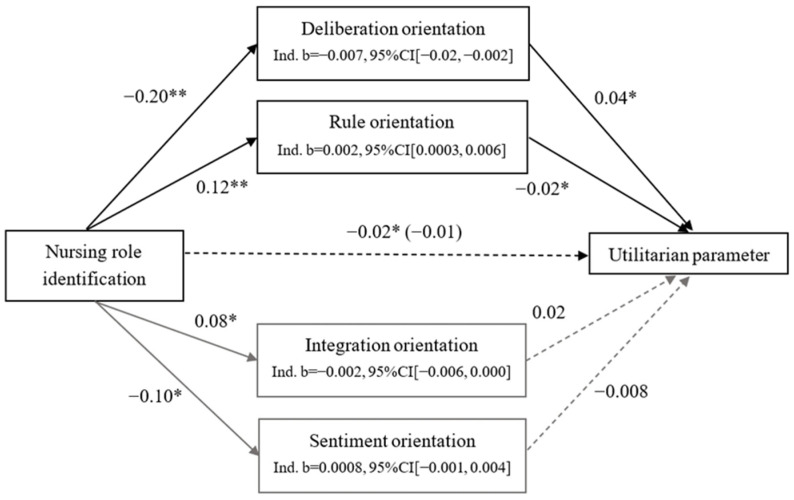
Mediation model for utilitarian (U) parameter. Note. * *p* < 0.05. ** *p* < 0.01.

We used Process for SPSS (Model 4 [44]) to examine whether each of the four moral orientations mediated the effect of nursing-role identification on the utilitarian parameter with 5000 bootstrapped samples. Only deliberation mediated the effect of nursing-role identification on utilitarian inclination (see Figure 1). Firstly, nursing-role identification was negatively associated with the utilitarian parameter, *b* = −0.02, SE = 0.007, *t*(495) = −2.21, *p* = 0.027, 95%CI [−0.03, −0.002]. Meanwhile, nursing-role identification negatively predicted deliberation, *b* = −0.13, SE = 0.03, *t*(495) = −5.18, *p* < 0.001, 95%CI [−0.18, −0.08], and deliberation was positively related with the utilitarian parameter, *b* = 0.03, SE = 0.01, *t*(495) = 1.99, *p* = 0.047, 95%CI [0.003, 0.06]. After including deliberation orientation, nursing-role identification had no relationship with the utilitarian parameter, *b* = −0.01, SE = 0.007, *t*(495) = −1.86, *p* = 0.06, 95%CI [−0.03, 0.0007]. Nursing-role identification was associated with a decreased utilitarian parameter because it was related to decreased deliberation orientation. However, nursing-role identification was not related with the rule, *b* = −0.06, SE = 0.03, *t*(495) = −1.70, *p* = 0.09, 95%CI [−0.13, 0.01], sentiment, *b* = −0.01, SE = 0.04, *t*(495) = −0.32, *p* = 0.75, 95%CI [−0.09, 0.06], and integration orientations, *b* = 0.02, SE = 0.03, *t*(495) = 0.76, *p* = 0.44, 95%CI [−0.04, 0.08]. Therefore, these three orientations did not mediate the relationship between the identification with nursing role and the utilitarian parameter.

**Figure 2:**
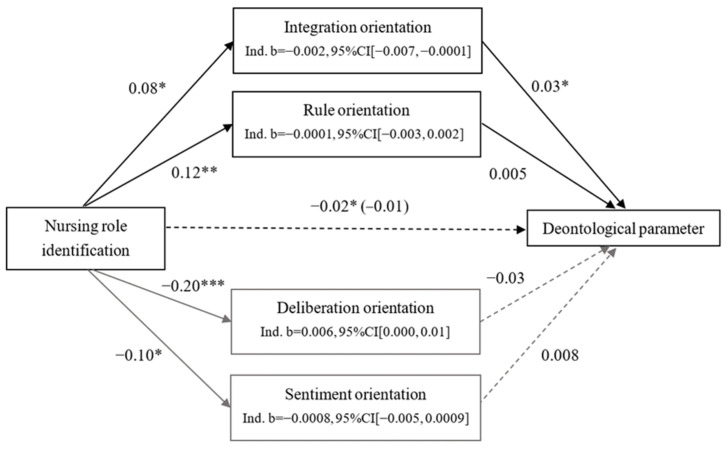
Mediation model for deontological (D) parameter. Note. * *p* < 0.05. ** *p* < 0.01. *** *p* < 0.001.

We also examined whether each of the four moral orientations mediated the effect of nursing-role identification on the deontological parameter with 5000 bootstrapped samples. Only deliberation orientations partly mediated the effect of nursing-role identification on deontological inclination (see Figure 2). Firstly, nursing-role identification was positively associated with the deontological parameter, *b* = 0.030, SE = 0.007, *t*(495) = 4.44, *p* < 0.001, 95%CI [0.017, 0.044]. Meanwhile, nursing-role identification negatively predicted deliberation orientation, *b* = −0.13, SE = 0.03, *t*(495) = −5.18, *p* < 0.001, 95% CI [−0.18, 0.08], and deliberation was negatively related to the deontological parameter, *b* = −0.03, SE = 0.02, *t*(495) = −2.17, *p* = 0.03, 95%CI [−0.06, −0.003]. After including deliberation orientation, the effect of nursing-role identification on the deontological parameter decreased, *b* = 0.025, SE = 0.007, *t*(495) = 3.53, *p* = 0.0004, 95%CI [0.011, 0.039]. This proves that deliberation partly mediated the effect of nursing-role identification on deontological inclination. As nursing-role identification was not related to the rule, sentiment, and integration orientations, these three orientations did not mediate the relationship between the identification with nursing role and the deontological parameter.

Study 1 used the scrambled-sentence task to prime nursing role, while Study 2 selected nursing-role identification, a trait variable, to investigate the effect of nursing role on moral judgments. Consistent with Study 1, Study 2 found that nursing role was positively associated with individuals’ deontological inclination. Nursing-role identification was negatively related to utilitarian inclinations, which is different from the effect of priming nursing role on moral judgments. Also, Study 2 explored the mechanism underlying the effect of nursing role on deontological and utilitarian thinking. Nursing role can decrease utilitarian moral thinking and increase deontological thinking because the identification with nursing role grants individuals a lower deliberation orientation.

## 4. General Discussion

Although a large number of previous studies have explored the moral judgment of nurses, most of the research has focused on the developmental stages of nurses’ moral levels and the association between moral judgment and their professional behavior [1,2,4]. These studies aimed to improve nurses’ moral development through educational interventions in order to enhance their ability to provide better healthcare services. However, there is limited research on how nurses engage in moral reasoning based on different moral principles when facing moral dilemmas. Deontology and utilitarianism are two moral frameworks that have received significant attention in the study of moral dilemmas. However, previous researchers have paid little attention to these aspects in the study of nurses’ moral judgment.

The current research examined how nursing role (the concept of nursing role and nursing-role identification) affects deontological and utilitarian moral thinking inclinations in moral judgments using the process dissociation technique. We predicted that both priming the concept of nursing role and nursing-role identification would decrease utilitarian inclinations and increase deontological moral inclinations in moral judgments. Also, we tested whether the effect of nursing role on deontological and utilitarian inclinations is mediated by moral orientations.

Our predictions are partly supported by two studies while activating the concept of nursing role and assessing nursing-role identification show different effects on moral judgments. Study 1 examined the effect of nursing role on moral judgments by activating the concept of nursing role, demonstrating that participants primed with nursing role showed higher deontological inclinations in moral judgments than those primed with a neutral concept. No difference was found in utilitarian moral thinking inclination between the two conditions. To test whether long-term attitudes towards the nursing profession have similar influences on moral judgments, Study 2 further tested the effect of nursing-role identification, and revealed that the nursing-role identification was positively related to deontological inclinations, and was negatively related to utilitarian inclinations, which was different from Study 1. We also found the mediating effect of deliberation underlying the relationship between nursing-role identification and deontological and utilitarian inclinations in Study 2.

As all the participants recruited for our study were adult nurses who were already working, we believe that their moral development levels have already reached a relatively high stage in Kohlberg’s theory [7] of moral development. Therefore, unlike previous research, our focus is not on how high individuals’ moral development levels are, but rather on how the nursing profession influences individuals’ moral reasoning and judgment after nurses have reached a relatively mature level of moral development. The nursing profession has its own uniqueness compared to other professions. Nurses are generally perceived to possess a higher morality level. Due to the connection between nursing work and human life and health, nurses bear significant responsibilities and are required to have a stronger sense of duty, as well as greater compassion and empathy compared to other professions [33,40]. Additionally, the demanding and high-pressure work environment may lead nurses to exhibit different moral judgment patterns compared to ordinary individuals or other professions in moral dilemmas [33]. Therefore, we believe that the nursing profession and the identification with nursing role can influence individuals’ moral judgments.

Nursing, as a profession, is characterized by a strong emphasis on ethical principles and codes of conduct [33]. Nurses are guided by professional ethics, such as beneficence, non-maleficence, autonomy, and justice [40], which align closely with deontological ethics. Furthermore, professional identification fosters a sense of responsibility and identification towards the nursing profession’s values and standards [34]. Nurses who deeply identify with their profession are more likely to perceive their role as one of moral stewardship and advocate for ethical practices [31]. They may prioritize upholding professional norms and obligations, even when faced with conflicting considerations or potential negative consequences. Deontological ethics prioritize adherence to moral rules and duties, regardless of the consequences or outcomes [12,13]. Nurses who strongly identify with their profession are more likely to internalize and embrace these ethical principles, leading to a higher inclination towards deontological reasoning.

In recent years, nurses have faced increasing workloads and time constraints [45]. Due to staffing shortages and an ever-increasing demand for healthcare services, nurses often find themselves working in a fast-paced and high-pressure environment [46]. As a result, they may not have enough time to engage in extensive deliberation when making decisions. The decrease in deliberation orientation can have implications for ethical decision-making as it can lead to higher deontological thinking. Deontological ethics emphasizes following moral rules and duties regardless of the consequences [13]. In the context of nursing, this means that nurses may rely more on established protocols and guidelines rather than considering the specific needs and values of individual patients.

Nevertheless, deliberation orientation only partially mediates the effect of nursing-role identification on deontological thinking. This means that there are still other mechanisms that can explain the relationship between nursing-role identification and deontological thinking. Further study can discuss this question.

Nursing-role identification reduces individuals’ utilitarian inclination, primarily due to nurses facing limited time and heavy workloads [33,45], which leaves them with insufficient time for deep reflection, while utilitarian moral tendencies often result from thoughtful deliberation. Nurses are not given ample time or opportunity to deliberate or think deeply about their actions and decisions. This decrease in deliberation orientation can lead to lower utilitarian thinking among nurses.

The nursing role guides people to not rely on utilitarian thinking to make judgments. However, this effect only exists in nursing-role identification and was not found in people primed with the role of nurse. Priming the nursing role and professional identification have different influences on moral reasoning. This suggests that the effects of word scramble tasks on moral judgments are different from long-term professional identification.

### 4.1. Practical Implication

This research contributes to the understanding of the moral thinking of nurses. When nurses think of and identify with their nursing profession, their deontological moral thinking increases, which helps them to be more responsible and compliant with professional standards in medical work, and they also become more concerned about the lives and health of patients. This provides some insights for the training of nurses and the education of nursing students. Hospitals and schools can enhance nurses’ professional identity education and training, which can increase their sense of responsibility and care for patients in their work practice.

While deontological thinking has its merits, it may overlook the unique circumstances and preferences of patients. It can result in a one-size-fits-all approach to care, potentially compromising patient-centeredness and autonomy. Utilitarian thinking is a moral framework that focuses on maximizing overall happiness or utility for the greatest number of people [12,13]. In the context of nursing, utilitarian thinking involves making decisions and taking actions that prioritize the well-being and best interests of patients. When nurses have less time for deliberation or thoughtful consideration, they may be more likely to rely on quick, instinctive judgments or follow established protocols without fully considering the unique needs and circumstances of individual patients. Furthermore, decreased deliberation orientation may limit nurses’ ability to critically analyze complex situations, weigh potential risks and benefits, and consider alternative courses of action. This can lead to suboptimal decision-making and potentially compromise patient outcomes. Therefore, it is important for nurses and healthcare organizations to strike a balance between adhering to guidelines and incorporating ethical deliberation to ensure the best possible care for patients.

### 4.2. Limitation and Future Research

The study has several limitations: First, due to the use of experimental methods, Study 1 is relatively time-consuming, making it difficult to collect data, resulting in a relatively small sample size of only 90 participants. Future research should consider validating this effect in a larger sample. Second, in the current study, we did not consider more detailed characteristics of the study population, such as work experience, position, or the specific professional field of nurses. These occupational factors may potentially influence their moral reasoning. For example, nurses with longer work experience may have more mature moral reasoning [47]. Future research should take these variables into consideration. Also, previous research has shown that people’s moral thinking varies across different cultures [48], and future studies can consider verifying the findings in different cultural contexts.

## 5. Conclusions

When nurses are primed with concepts related to their profession, their deontological moral inclinations are elevated compared to when they are primed with neutral concepts. Nurses’ professional identification is positively associated with their deontological inclinations, while it is negatively associated with utilitarian inclinations. This association is due to the decreased tendency for deliberation.

## Data Availability

The raw data supporting the conclusions of this article will be made available by the authors upon request.
